# HOMA-IR as a Predictor of PAI-1 Levels in Women with Severe Obesity

**DOI:** 10.3390/biomedicines12061222

**Published:** 2024-05-31

**Authors:** Fabiana Martins Kattah, Milijana Janjusevic, Nayra Figueiredo, Emilly Santos Oliveira, Glaucia Carielo Lima, Ana Raimunda Dâmaso, Lila Missae Oyama, Alessandra Lucia Fluca, Paulo Reis Eselin de Melo, Maria Aderuza Horst, Aneta Aleksova, Flávia Campos Corgosinho

**Affiliations:** 1 Faculty of Nutrition, Federal University of Goias, Goiânia 74605-080, Brazil; emillysantos@discente.ufg.br (E.S.O.); glauciacarielo@ufg.br (G.C.L.); aderuza@ufg.br (M.A.H.); 2 Cardiothoracovascular Department, Azienda Sanitaria Universitaria Giuliano Isontina (ASUGI), Department of Medical Surgical and Health Science, University of Trieste, 34149 Trieste, Italy; mjanjusevic@units.it (M.J.); alessandralucia.fluca@units.it (A.L.F.); or aaleksova@gmail.com (A.A.); 3 Faculty of Medicine, Federal University of Goias, Goiania 74605-080, Brazil; nayrafgrdo@hotmail.com; 4 Paulista Medicine School, Federal University of São Paulo, São Paulo 04023-062, Brazil; ana.damaso@unifesp.br (A.R.D.); lmoyama@unifesp.br (L.M.O.); 5 Alberto Rassi Hospital, Goiânia 74110-010, Brazil; prcirurgia@icloud.com

**Keywords:** PAI-1, cardiovascular disease, inflammation, severe obesity, HOMA-IR

## Abstract

Background: Obesity is a chronic inflammatory disorder that increases the risk of cardiovascular diseases (CVDs). Given the high CVD mortality rate among individuals with obesity, early screening should be considered. Plasminogen activator inhibitor (PAI-1), a cytokine that links obesity and CVDs, represents a promising biomarker. However, PAI-1 is not part of the clinical routine due to its high cost. Therefore, it is necessary to find good predictors that would allow an indirect assessment of PAI-1. Methods: This study enrolled 47 women with severe obesity (SO). The obtained anthropometric measurements included weight, height, neck (NC), waist (WC), and hip circumference (HC). Blood samples were collected to analyse glucose and lipid profiles, C-reactive protein, liver markers, adiponectin, and PAI-1 (determined by ELISA immunoassay). Homeostasis model assessment-adiponectin (HOMA-AD), homeostasis model assessment of insulin resistance (HOMA-IR), quantitative insulin sensitivity check index (QUICKI), triglyceride–glucose index (TyG), and atherogenic index of plasma (AIP) were calculated. The women were grouped according to PAI-1 levels. The data were analysed using IBM SPSS Statistics, version 21. The significance level for the analysis was set at 5%. Results: Women with SO who have higher levels of PAI-1 have lower values of high-density lipoprotein cholesterol (HDL) (*p* = 0.037) and QUICKI (0.020) and higher values of HOMA-AD (0.046) and HOMA-IR (0.037). HOMA-IR was demonstrated to be a good predictor of PAI-1 in this sample (B = 0.2791; *p* = 0.017). Conclusions: HOMA-IR could be used as a predictor of PAI-1 levels, pointing out the relevance of assessing glycaemic parameters for the prevention of CVDs in women with SO.

## 1. Introduction

Obesity, a health-threatening condition characterised by an excessive accumulation of body fat [1], is increasing in prevalence worldwide across all ages, genders, nationalities, and socioeconomic status [2]. Obesity is associated with an increase in cardiometabolic risk through effects on cardiovascular structure, the promotion of a pro-inflammatory state with alterations in cytokine secretion patterns, and the emergence of other metabolic disorders [3]. More precisely, obesity is associated with a worse lipid panel, increased blood pressure, impaired plasma glucose levels, type 2 diabetes mellitus (T2DM), liver dysfunction, and low levels of cardiorespiratory fitness parameters, factors that contribute to cardiovascular diseases (CVDs) [4].

One of the cytokines whose expression is elevated in obesity and increases the risk of CVDs is the plasminogen activator inhibitor (PAI-1), a regulator of fibrinolysis that acts on thrombogenic pathways [5]. The main activity of PAI-1 is to inhibit both the tissue and urokinase plasminogen activators, which are responsible for the cleavage of plasminogen to plasmin [6]. The processes of fibrinogenesis and fibrinolysis are important in both intravascular and extravascular physiology and the pathology of CVDs [7]. However, the role of PAI-1 is not limited to the control of fibrinolysis, as it is also involved in the control of tissue remodelling, angiogenesis, inflammation, and extracellular matrix degradation [6]. By controlling this plethora of mechanisms, PAI-1 has previously been reported to participate in the pathophysiology of several metabolic syndromes, including obesity and insulin resistance. 

The interplay between PAI-1 and glycaemic control appears to be related to hyperinsulinemia promoting PAI-1 expression as well as reducing the rate of mRNA degradation of PAI-1, supporting protein production [8,9]. Furthermore, it has been hypothesised that insulin resistance decreases the activity of the PI3-K/Akt pathway while upregulating the mitogen-activated protein kinase/extracellular signal-regulated kinase (MAPK/ERK) pathway, favouring the release of inflammatory markers, among them PAI-1 [10]. Also, given that individuals with atherosclerotic plaque have increased PAI-1 expression [11] and that PAI-1 concentrations are associated with the severity of subclinical atherosclerosis in patients with obesity, PAI-1 quantification may be an additional tool to identify patients with obesity at higher risk of developing CVDs [11].

However, despite PAI-1 being a good predictor, the high cost of measuring this adipokine prevents its use in routine clinical practice. Therefore, it is important to explore other parameters that can be predictors of PAI-1. The atherogenic index of plasma (AIP) is independently correlated with a higher incidence of coronary heart disease [12] and appears to be associated with PAI-1 levels in individuals with severe obesity; however, further studies are needed to confirm this possible relationship [11]. The triglyceride (TG)–high-density lipoprotein cholesterol (HDL-c) ratio, which is a component of the AIP equation, could be a parameter for the identification of individuals with severe obesity at risk of developing metabolic syndrome (MS) [13]. Furthermore, although the triglyceride–glucose index (TyG) is also used as a cardiometabolic risk marker and may be used as a marker of atherosclerosis [14], no studies have evaluated its correlation with PAI-1 levels. In addition, the homeostasis model assessment of insulin resistance (HOMA-IR) and quantitative insulin sensitivity check index (QUICKI) are usually performed to determine insulin resistance as a TyG, but little is known about the relationship between those markers and PAI-1 values as well. Thus, this study aimed to verify the correlation of PAI-1 with cardiometabolic risk markers in women with severe obesity, seeking to evaluate predictors of PAI-1 levels. Thus, the hypothesis of this study is that some of those indexes can predict PAI-1 in order to better screen patients with higher cardiovascular risk. 

## 2. Material and Methods

### 2.1. Participants

The study population consisted of 47 women with a body mass index (BMI) above 40 kg/m^2^ (severe obesity) who were hospitalised for bariatric surgery at the Hospital Estadual Geral de Goiânia Dr Alberto Rassi (HGG), Goiânia, GO, Brazil. BMI was calculated by dividing the person’s weight by the square of their height (in metres) [15]. The research team obtained a list of patients eligible for bariatric surgery from the Obesity Surgery Control Program (PCCO). During the first outpatient consultation at the HGG, the researcher explained the aim of the project to patients who met the inclusion criteria, and informed consent forms were signed in duplicate. Data collected for this study included age, date of birth, medication use, the presence of comorbidities such as T2DM, hypertension, thyroid dysfunction, and anthropometric measurements. In addition, blood samples were collected from all participants.

Non-inclusion criteria were participants younger than 20 years or older than 59 years with acute inflammatory, infectious, or neoplastic diseases, genetic syndromes, rheumatic and autoimmune diseases, fibromyalgia, chronic alcohol consumption (>30 g/d), or abuse of illicit/psychotropic drugs. 

This study was carried out according to the principles of the Declaration of Helsinki and was approved by the Ethics Committees of the Universidade Federal de Goiás and the hospital (3,251,178 and 961/19, respectively).

### 2.2. Anthropometric Measurements

The anthropometric assessment was given by the mean value of two measurements of weight, height, hip, waist, and neck circumference. Weight was measured using the Lider scale with a maximum capacity of 200 kg, with the volunteer standing in the centre of the scale wearing light clothes and no shoes. Height was measured with the patient in an upright position, barefoot, looking forward, and with arms outstretched at the sides, using a Fillizola scale available at the hospital. BMI was calculated by dividing the person’s weight by the square of their height (in metres), and obesity was classified as grade I (30 to 34.99 kg/m^2^), grade II (35 to 39.99 kg/m^2^), and grade III (≥40 kg/m^2^). The waist circumference was measured at the level of the umbilical line while the volunteer was standing. The neck circumference was measured below the level of the cricoid cartilage. All measurements were taken by a trained researcher. 

### 2.3. Blood Analysis

Blood sampling was performed by peripheral vein puncture of the forearm by trained nurses after a 12 h overnight fast. The collection was carried out in the laboratory of Atalaia Medicina Diagnóstica, Goiânia—GO. Biochemical analysis was performed with a colorimetric enzymatic method, specific for each dose (insulin, blood glucose, glycated haemoglobin A1c (HbA1c), lipid profile, and ultra-high sensitive C-reactive protein (hs-CRP), according to the laboratory). For additional analysis, EDTA tubes containing samples from each participant were transported in a thermal box to the Clinical Nutrition Research Laboratory and Sports (LABINCE), located at the Faculty of Nutrition (FANUT) of the Federal University of Goiás (UFG). After centrifugation, the serum was stored at −80 °C until use. PAI-1 and adiponectin levels were determined by enzyme-linked immunosorbent assay (ELISA) using a commercial kit (R&D Systems, Minnesota, EUA) according to the manufacturer’s instructions performed at the Laboratory of Nutrition Physiology of the Federal University of São Paulo (UNIFESP). 

### 2.4. Index Calculation

The AIP was calculated from the logarithm of the triglyceride–high-density lipoprotein cholesterol ratio (TG/HDL-c ratio) [12]. The HOMA-IR was obtained with the formula: fasting insulin (µUI/L) × blood glucose (mg/dL)/22.5 [16]. The homeostasis model assessment-beta (HOMA-beta) and QUICKI were calculated from blood glucose and insulin values as reported in the literature [16,17]. The homeostasis model assessment-adiponectin (HOMA-AD) was calculated as the product of fasting insulin (µUI/L) and blood glucose (mg/dL), divided by adiponectin (mg/mL) [18]. TyG was calculated by Ln (fasting triglycerides (mg/dL) × fasting blood glucose (mg/dL)/2) [19]. As there is no reference value for PAI-1 in severe obesity in the literature, the median value of PAI-1 in our cohort (21 ng/mL) was used for study purposes. Values less than 4 µg/mL for adiponectin were considered hypoadiponectinemia [20].

### 2.5. Statistical Analysis

Statistical analysis was performed using the Statistical Package for the Social Sciences (SPSS), version 24.0. The data were first assessed for normality using the Shapiro–Wilk test. Non-normally distributed variables were standardised using the Z-score and are presented as the mean ± standard deviation. Volunteers were grouped according to the median PAI-1 value, as is usually performed when there is a lack of a cut-off literature value for the marker of interest [21,22]. Pearson’s or Spearman’s correlation analysis was performed to assess correlations between the studied variables, as appropriate. To compare the difference in means, the *t*-test for independent samples was used. The chi-square test was performed to compare the frequencies of pathologies in the groups. A bivariate logistic regression model was performed after the comparison of averages. The calculation of post hoc sample power was performed using GPower (version 3.0) to determine the effect size. We considered a binomial distribution, an odds ratio of 1.32; Pr(Y = 1) H0: 0.05; α err prob: 0.05; R^2^ other X: 0.232; X parm π of 0.5; and a total sample of 47 individuals, obtaining a sample power of 5.26%. A *p* value ≤ 0.05 was considered statistically significant.

## 3. Results

### 3.1. Characteristics of the Population

Forty-seven women were enrolled in this study. At baseline, the mean age was 40 ± 8.3 years old, and all volunteers had a BMI compatible with severe obesity. Neck and abdominal circumferences were higher than recommended, indicating a high cardiovascular risk. Moreover, the mean blood pressure classifies this sample as hypertension stage 1 [23]. Mean blood glucose values, insulin, HOMA-IR, HOMA-beta, and HbA1c also indicate the presence of cardiometabolic risk and insulin resistance. The mean AIP was 0.47 ± 0.21. The baseline characteristics of the patients are presented in Table 1.

The cytokine PAI-1 did not correlate with any variable in the overall sample. AIP correlated with TG (r = 0.904, *p* < 0.0001), HDL-c (r = −0.494, *p* = 0.001), very-low-density lipoprotein (VLDL) (r = 0.900, *p* < 0.0001), alanine aminotransferase (ALT) (r = 0.522, *p* < 0.0001), adiponectin (r = −0.352, *p* = 0.019), and TyG (r = 0.792, *p* < 0.0001) (Table 2). 

Furthermore, TyG correlated with blood glucose (r = 0.547, *p* < 0.0001), HOMA-beta (r = −0.461, *p* = 0.002), TG (r = 0.833, *p* < 0.0001), ALT (r = 0.522, *p* < 0.0001), HbA1c (r = 0.352, *p* = 0.019), estimated average glucose (EAG) (r = 0.446, *p* = 0.003), adiponectin (r = −0.431, *p* = 0.004), QUICKI (r = −0.384, *p* = 0.012), and HOMA-AD (r = 0.363, *p* = 0.020) (Table 3).

When stratifying individuals based on median PAI-1 concentration (21 ng/mL), patients with higher PAI-1 values had significantly higher HOMA-IR (*p*= 0.037, Cohen’s d = −0.67) and HOMA-AD (*p* = 0.046, Cohen’s d = −0.65) levels compared to the individuals with lower PAI-1 blood concentration, while the QUICKI (*p*= 0.022, Cohen’s d = 0.75) and HDL-c (Cohen’s d = 0.64) were significantly lower in the same group, indicating worse cardiometabolic and insulin features, as shown on Table 1.

There was no difference between the groups for the incidence of hypertension, T2DM, hypercholesterolemia, thyroidopathy, insulin resistance, and hypoadiponectinemia. When comparing groups based on the use of hypoglycaemic and antihypertensive drugs, we did not observe any difference between the groups in terms of PAI-1 levels (Table S1). 

In the group with a higher PAI-1 value, there was a positive correlation between PAI-1 and hs-CRP (r = 0.674, *p* = 0.002) (Table 4). In this group, AIP remained correlated only with ALT (r = 0.517, *p* = 0.020).

It was possible to observe a negative correlation between TyG and QUICKI only in the group with lower PAI-1 values (r = −0.459, *p* = 0.032) (Table 5).

### 3.2. PAI-1 Predictors

In the regression analysis, it was found that there is an additional risk of 32.1% of belonging to the group with higher cardiovascular risk (higher PAI-1) with an increase in one unit in HOMA-IR. The results showed HOMA-IR as a predictor of PAI-1 (*p* = 0.017) (Table 6).

## 4. Discussion

It is beyond doubt that inflammation plays a pivotal role in the onset of obesity and the development of cardiovascular diseases. The role of PAI-1 in inflammation and CVDs is well established; however, it is important to note that the analysis of PAI-1 levels can be challenging and expensive, which further contributes to the limited use of this important marker in clinical practice. For the first time, we were able to show HOMA-IR, a very common biochemical marker, as a predictor of PAI-1 in women with severe obesity. A previous study by Basurto et al. [24], which included individuals with normal weight, overweight, and subjects with obesity, observed the influence of HOMA-IR on PAI-1 concentration; however, the study did not include individuals with a BMI > 40 kg/m. 

In addition to the hypothesis that higher PAI-1 values are promoted by hyperinsulinemia and insulin resistance [8,9,10], the relationship between PAI-1 and glycaemia is also demonstrated by the effects of common hypoglycaemic drugs such as pioglitazone, troglitazone, and metformin, which reduce serum levels of PAI-1 [25,26,27] as well as its activity [28]. We did not find a statistically significant effect of glucose-lowering drugs on PAI-1 levels in our cohort. Nevertheless, controlling PAI-1 levels through pharmacological intervention could have multifactorial beneficial effects for patients with obesity-related T2DM. 

In fact, the present study demonstrated that women with severe obesity and higher levels of PAI-1 presented lower values of QUICKI and higher levels of HOMA-AD and HOMA-IR, reinforcing the relationship between PAI-1 and glycaemic traits and emphasising its importance in clinical practice. Corroborating with our data, previous studies have demonstrated the relationship between PAI-1 and glycaemic metabolic parameters [24,29,30], showing that metabolically unhealthy individuals have higher levels of PAI-1 independently of BMI [24]. Mendivil et al. [29] observed a positive correlation between PAI-1 levels and insulin resistance parameters in subjects at high risk of developing T2DM. In addition, a study of 295 individuals aged between 18 and 45 years, including eutrophic, overweight, and patients with obesity (without a diagnosis of T2DM or hypertension), found that PAI-1 was a negative predictor of QUICKI [30]. The exact mechanisms that precisely define PAI-1 involvement in glycaemic control are not clear yet [31]. Some hypotheses postulate that PAI-1 has deleterious effects on some proteins, such as the insulin receptor, transforming growth factor beta, and peroxisome proliferator-activated receptor gamma γ, promoting insulin resistance [32]. Therefore, more studies are needed to elucidate the underlying mechanisms. 

Although we did not observe significant differences in adiponectin levels between the groups stratified according to low and high PAI-1 levels, we did observe that patients with higher PAI-1 presented higher HOMA-AD, an important index combining insulin resistance and adiponectin. A study including individuals with T2DM and MS observed that PAI-1 mediates the downregulation of adiponectin [33]. Changes in adiponectin levels can alter the insulin signalling cascade, as adiponectin binds to leucine zipper 1 (APPL1) [34].

It is important to note that PAI-1 can also contribute to CVDs by altering cholesterol homeostasis [35]. In fact, our study showed that individuals with higher PAI-1 values had lower HDL-c levels, a lipoprotein with cardioprotective effects. Corroboratingly, Basurto et al. evaluated individuals based on metabolic assessment and observed a negative correlation of PAI-1 with HDL-c in women with normal weight, overweight, and obesity [24]. In addition, a systematic review and meta-analysis also suggested a causal effect of PAI-1 on HDL-c [36]. Together, our data support the importance of PAI-1 in cardiovascular health by influencing both the glycaemic and lipid markers related to endothelial dysfunction. 

Moreover, it is well established that high levels of PAI-1 and hs-CRP are associated with insulin resistance and microvascular dysfunction and may contribute to CVDs [30]. Our data pointed out that in the group of patients with a higher PAI-1 value, there is a strong and positive correlation between PAI-1 and hs-CRP. A correlation between PAI-1 and CRP has previously been observed in individuals with DM, demonstrating the association between these markers in individuals with DM and carriers of the 4G polymorphism in the PAI-1 gene [37]. 

Regarding other cardiometabolic indexes, high mean AIP values (0.47 ± 0.21) were observed in our cohort, which also supports the increased cardiovascular risk being defined as AIP > 0.21 by Holmes et al. [38]. However, we did not find any associations between PAI-1 and AIP and TyG, which could be explained by the small sample size, as we expected given the data from a study of individuals with severe obesity that demonstrated an association between PAI-1 and AIP [11]. 

Concerning the association between the TyG and PAI-1, to our knowledge, there are no studies investigating the topic, although the association between PAI-1 concentrations and high concentrations of glucose and triglycerides (parameters of the index) has been demonstrated [24]. Nevertheless, we were able to show a positive correlation between TyG and HOMA-AD, and this correlation remained significant only in the group with higher values of PAI-1. We also observed a negative correlation between TyG and QUICKI, parameters that evaluate insulin resistance and sensibility, respectively, in the group with lower levels of PAI-1, reinforcing PAI-1 as a factor influencing glycaemic parameters. In addition, we demonstrated the correlations between AIP and TyG, which represent a cardiovascular risk factor.

Finally, our data showed a positive correlation between AIP and ALT. This finding is consistent with a large-scale study conducted in China with 7838 participants that showed AIP to be an independent risk predictor for fatty liver [39]. PAI-1 expression is known to be significantly higher in patients with non-alcoholic fatty liver disease (NAFLD), suggesting that NAFLD independently contributes to PAI-1 secretion [40]. Furthermore, an association has been found between plasma levels of PAI-1 and the ALT/aspartate aminotransferase (AST) ratio in individuals with severe obesity [41]. Corroboratingly, our study showed a correlation of AIP with ALT only in the group with high PAI-1 values, suggesting that clinicians should recognise the increased risk of CVDs in NAFLD patients. 

This is the first study comparing women with severe obesity based on PAI-1 levels. It is important to note that there is a lack of PAI-1 cut-off value in the literature, which led us to group the patients according to the median values, as previously performed when no cut-off value had been established. Taking together our findings, we suggest that in a homogeneous sample (no differences in anthropometric assessments), individuals with higher levels of PAI-1 had a worse cardiometabolic profile, and HOMA-IR might be a useful tool to screen patients with higher PAI-1. The limitations of this study are the cross-sectional design, small sample size, and absence of a control group. Further studies with a larger sample size, especially in severe obesity, are needed to confirm these results and set up a cut-off value for this important cytokine.

## 5. Conclusions

Women with severe obesity and higher PAI-1 levels have an increased cardiometabolic risk, as indicated by higher HOMA-IR and HOMA-AD values, lower QUICKI, and lower HDL-c concentrations. Finally, HOMA-IR could be used as a predictor of PAI-1 levels, highlighting the importance of assessing glycaemic parameters in the prevention of CVDs in women with severe obesity. 

## Figures and Tables

**Table 1:** Descriptive analyses of women with severe obesity.

Variable	Total Sample	The group with Lower Levels of PAI-1 (≤21 ng/mL)	The Group with Higher Levels of PAI-1 (>21 ng/mL)	*p*-Value
Age (years)	40.08 ± 8.30	41.64 ± 8.27	38.46 ± 8.31	0.186
Height (m)	1.59 ± 0.06	1.58 ± 0.06	1.60 ± 0.06	0.276
Weight (kg)	122.68 ± 18.86	123.84 ± 14.98	121.57 ± 22.23	0.486
BMI (kg/m^2^)	48.23 ± 6.77	49.34 ± 5.87	47.16 ± 7.51	0.172
NC (cm)	41.58 ± 3.18	41.68 ± 3.28	41.88 ± 0.16	0.832
WC (cm)	130.77 ± 12.38	132.41 ± 10.46	129.17 ± 14.02	0.376
HC (cm)	144.09 ± 13.22	145.00 ± 11.64	143.20 ± 14.77	0.480
Fasting Glucose (mg/dL)	117.72 ± 50.57	110.39 ± 37.42	126.15 ± 62.38	0.312
Insulin (µUI/mL)	26.36 ± 12.76	23.33 ± 10.76	29.70 ± 14.17	0.107
HOMA-IR	7.37 ± 3.90	6.19 ± 3.51	8.65 ± 4.06	0.037 *
HOMA-beta	262.18 ± 182.90	240.36 ± 132.3	286.18 ± 184.5	0.697
QUICKI	0.295 ± 0.022	0.303 ± 0.023	0.287 ± 0.019	0.020 *
HOMA-AD	472.21 ± 368.02	362.07 ± 274	587.87 ± 422.6	0.046 *
TG (mg/dL)	145.34 ± 67.14	140.75 ± 49.8	150.85 ± 84.5	0.482
TC (mg/dL)	176.14 ± 31.28	179.42 ± 31.5	172.20 ± 49, 31.2	0.453
HDL-c (mg/dL)	45.86 ± 9.78	48.58 ± 9.87	42.60 ±8.82	0.042 *
LDL-c (mg/dL)	105.01± 28.65	105.90 ± 31.9	103.95 ± 24.9	0.811
VLDL-c (mg/dL)	25.26 ± 8.80	24.79 ± 6.51	25.80 ± 11.02	0.568
ALT (U/L)	18.12 ± 8.23	15.86 ± 5.80	20.60 ± 9.83	0.062
AST (U/L)	17.43 ± 7.03	15.77 ± 4.09	19.25 ± 9.02	0.108
hsCRP (mg/dL)	1.28 ± 1.05	1.34 ± 0.95	1.20 ± 1.16	0.653
HbA1c (%)	6.37 ± 1.37	6.26 ± 1.30	6.49 ± 1.47	0.627
EAG (mg/dL)	136.09 ± 39.40	133.18 ± 37.35	139.30 ± 42.27	0.621
Adiponectin (µg/mL)	7.84 ± 5.25	8.60 ± 6.69	7.05 ± 3.10	0.245
PAI-1 (ng/mL)	22.95 ± 12.09	15.58 ± 4.44	27.24 ± 5.36	<0.0001 *
AIP	0.47 ± 0.21	0.44 ± 0.19	0.50 ± 0.23	0.389
TyG	4.16 ± 0.24	4.14 ± 0.20	4.19 ± 0.27	0.547
Systolic Blood Pressure	140.46 ± 15.5	139.42 ± 16.14	141.5 ± 15.3	0.731
Diastolic Blood Pressure	87.67 ± 9.55	87. 42 ± 9.12	87.9 ± 10.3	0.931
Pathology (%)	72.3	76	68.2	0.550
Hypertension (%)	55.4	56	54.5	0.920
T2DM (%)	23.4	16	31.8	0.201
Thyroidopathy (%)	12.8	84	90.9	0.479
Insulin Resistance (%)	78.7	76	81.8	0.368

AIP: atherogenic index of plasma (≤0.21); ALT: alanine aminotransferase (<33 U/L); AST: aspartate aminotransferase (< 32 U/L); BMI: body mass index; EAG: estimated average glucose; adiponectin (>4 µg/mL); HbA1c: glycated haemoglobin (>5.7%); HC: hip circumference; glucose (between 70 and 99 mg/dL); HDL-c: high-density lipoprotein (>40 mg/dL); HOMA-AD: homeostasis model assessment-adiponectin (>0.504); HOMA-beta: homeostasis model assessment of β-cell function (154 ± 73); HOMA-IR: homeostasis model assessment of insulin resistance (<2.71); hs-CRP: ultra-high sensitive C-reactive protein (<1 mg/dL); insulin (between 2.6 and 24.9 μIU/mL); LDL-c: low-density lipoprotein (<130 mg/dL); NC: neck circumference (≤34 cm); PAI-1: plasminogen activator inhibitor-1; QUICKI: quantitative insulin sensitivity check index (≥0.321); T2DM: type 2 diabetes mellitus; TC: total cholesterol (<190 mg/dL); TG: triglycerides (<150 mg/dL); TyG: triglyceride–glucose index (<4.55); VLDL-c: very-low-density lipoprotein; WC: waist circumference (<80 cm). * *p* ≤ 0.05.

**Table 2:** Correlations between AIP and metabolic variables in the total sample.

		TG	HDL-c	VLDL	ALT	Adiponectin	TyG
AIP	r	0.904	−0.494	0.900	0.522	−0.352	0.792
	*p*	<0.0001 *	0.001 *	<0.0001 *	<0.0001 *	0.019 *	<0.0001 *

AIP: atherogenic index of plasma (≤0.21); ALT: alanine aminotransferase; HDL-c: high-density lipoprotein; TG: triglycerides; TyG: triglyceride–glucose index (<4.55); VLDL-c: very-low-density lipoprotein. * *p* ≤ 0.05.

**Table 3:** Correlations between TyG and metabolic variables in the total sample.

		Glycaemia	HOMA-beta	TG	ALT	HbA1c	EAG	Adiponectin	HOMA-AD	QUICKI
TyG	r	0.547	−0.461	0.833	0.522	0.352	0.446	−0.431	0.363	−0.384
	*p*	<0.0001 *	0.002 *	<0.0001 *	<0.0001 *	0.019 *	0.003 *	0.004 *	0.020 *	0.012 *

ALT: alanine aminotransferase; EAG: estimated average glucose; HbA1c: glycated haemoglobin; HOMA-AD: homeostasis model assessment-adiponectin; HOMA-beta: homeostasis model assessment of β-cell function; QUICKI: quantitative insulin sensitivity check index; TG: triglycerides; TyG: triglyceride–glucose index (<4.55). * *p* ≤ 0.05.

**Table 4:** Significant correlations in the group with higher levels of PAI-1.

		hs-CRP	ALT	HOMA-beta	HOMA-AD
PAI-1	r	0.674	−0.216	−0.087	−0.063
	*p*	0.002 *	0.360	0.072	0.797
AIP	r	−0.337	0.517	−0.316	0.330
	*p*	0.147	0.020 *	0.188	0.155
TyG	r	0.054	0.531	0.659	0.470
	*p*	0.820	0.016 *	0.002 *	0.036 *

AIP: atherogenic index of plasma (≤0.21); hs-CRP: ultra-high sensitive C-reactive protein; ALT: alanine aminotransferase; HOMA-beta: homeostasis model assessment of β-cell function; HOMA-AD: homeostasis model assessment-adiponectin; TyG: triglyceride–glucose index (<4.55). * *p* ≤ 0.05.

**Table 5:** Significant correlations in the group with lower levels of PAI-1.

		QUICKI	Hs-CRP	HOMA-beta	HOMA-AD
TyG	r	−0.459	−0.145	−0.152	0.251
	*p*	0.032 *	0.519	0.500	0.271

HOMA-AD: homeostasis model assessment-adiponectin; HOMA-beta: homeostasis model assessment of β-cell function; hs-CRP: ultra-high sensitive C-reactive protein; QUICKI: quantitative insulin sensitivity check index; TyG: triglyceride–glucose index. * *p* ≤ 0.05.

**Table 6:** Bivariate logistic regression model for PAI-1 in women with severe obesity.

		B	*p*-Value	OR (95% for Exp (B))
Step 1	HDL-c	−0.052	0.256	0.95 (0.87–1.04)
	HOMA-AD	0.000	0.862	1 (0.996–1.004)
	QUICKI	−9.030	0.835	0.000 (0.000–1.03)
	HOMA-IR	0.247	0.431	1.280 (0.692–2.367)
	Constant	3.321	0.815	27.677
Step 2	HDL-c	−0.053	0.232	0.948 (0.869–1.035)
	QUICKI	−9.117	0.834	0.000 (0.00–9.15)
	HOMA-IR	0.217	0.404	1.243 (0.746–2.070)
	Constant	3.471	0.807	32.158
Step 3	HDL-c	−0.055	0.203	0.946 (0.869–1.030)
	HOMA-IR	0.266	0.027 *	1.305 (1.030–1.654)
	Constant	0.525	0.806	1.690
Step 4	HOMA-IR	0.279	0.017 *	1.321 (1.052–1.659)
	Constant	−2.069	0.018	0.126

HDL-c: high-density lipoprotein; HOMA-AD: homeostasis model assessment-adiponectin; HOMA-IR: homeostasis model assessment of insulin resistance; QUICKI: quantitative insulin sensitivity check index; Binomial logistic regression, * *p* ≤ 0.05.

## Data Availability

The original contributions presented in this study are included in this article and Supplementary Materials; further inquiries can be directed to the corresponding authors.

## Supplementary Materials

The following supporting information can be downloaded at: https://www.mdpi.com/article/10.3390/biomedicines12061222/s1, Table S1: Comparison between individuals based on treatment with hypoglycaemic and antihypertensive drugs.
