# Supplementary material for: HOMA-IR as a Predictor of PAI-1 Levels in Women with Severe Obesity
Source: Biomedicines. 2024 May 31;12(6):1222. doi: 10.3390/biomedicines12061222 (PMC11200361; doi:10.3390/biomedicines12061222)
Supplement: Supplementary file 1 [file biomedicines-12-01222-s001.zip › biomedicines-3002559-supplementary.pdf]

Table S1. Comparison between individuals based on treatment with hypoglycemic and antihypertensive drugs.

Group	PAI-1 values	p-value
Hypoglicemic drug	25.28 ± 6.45	0.062
Non-hypolicemic drug	20.03 ± 7.59	
Antihypertensive drug	19.87 ± 7.36	0.308
Non-antihypertensive drug	22.16 ± 7.84	
